# Valosin-containing protein Asp395Gly mutation in a patient with frontotemporal dementia: a case report

**DOI:** 10.1186/s12883-022-02951-4

**Published:** 2022-11-03

**Authors:** Ryota Kobayashi, Hiroya Naruse, Shinobu Kawakatsu, Chifumi Iseki, Yuya Suzuki, Shingo Koyama, Daichi Morioka, Hiroyuki Ishiura, Jun Mitsui, Yasuyuki Ohta, Shoji Tsuji, Tatsushi Toda, Koichi Otani

**Affiliations:** 1 Department of Psychiatry, Yamagata University School of Medicine, 2-2–2 Iidanishi, Yamagata, 990-9585 Japan; 2 Department of Neurology, Graduate School of Medicine, The University of Tokyo, Tokyo, Japan; 3 Department of Neuropsychiatry, Aizu Medical Center, Fukushima Medical University, Aizuwakamatsu, Japan; 4 Department of Neurology, Hematology, Metabolism, Endocrinology, and Diabetology, Yamagata University School of Medicine, Yamagata, Japan; 5 Department of Molecular Neurology, Graduate School of Medicine, The University of Tokyo, Tokyo, Japan; 6 Institute of Medical Genomics, International University of Health and Welfare, Chiba, Japan

**Keywords:** Depression, Familial FTD, Frontotemporal dementia, Frontotemporal lobar degeneration, FTLD-TDP, FTLD-tau, Neurofibrillary tau tangles, Tauopathy, Valosin-containing protein

## Abstract

**Background:**

Variants in the *valosin-containing protein (VCP)* gene were identified as one of the causes for inclusion body myopathy associated with Paget disease of the bone and frontotemporal dementia (FTD). Previously identified pathogenic variants in *VCP* are associated with frontotemporal lobar degeneration with TDP-43 inclusions (FTLD-TDP) pathologically, but p.Asp395Gly *VCP* was recently reported to cause familial FTD with tauopathy characterized by neurofibrillary tau tangles (NFT) and not FTLD-TDP. We describe the clinical and genetic findings of a patient with p.Asp395Gly *valosin-containing protein (VCP)*, who was diagnosed with FTD without a family history and in the absence of muscle or bone disease comorbidity.

**Case presentation:**

The patient was a 62-year-old man, who developed atypical depression at the age of 37 years. Subsequently, he presented with self-centered behavior at the age of 45 years. The self-centered behavior intensified from around the age of 50 years, which was accompanied by the development of executive dysfunction; therefore, he visited our hospital at 52 years of age. Magnetic resonance imaging revealed bilateral frontal lobe atrophy. Brain perfusion single-photon emission computed tomography revealed bilateral frontal lobe hypoperfusion. The patient fulfilled the diagnostic criteria for behavioral variant of FTD. Ten years after the diagnosis, computed tomography of the trunk and limbs, muscle biopsy, and bone scintigraphy revealed the absence of concomitant muscle and bone disease. The concentrations of cerebrospinal fluid (CSF) total tau and phosphorylated tau proteins were 389 pg/mL and 53.2 pg/mL (cut-off: 50 pg/mL), respectively. Genetic analyses were performed using the whole-exome and Sanger sequencing methods. We identified p.Asp395Gly *VCP* in this patient with pure FTD.

**Conclusions:**

p.Asp395Gly *VCP* was identified in a patient with likely sporadic FTD without concomitant muscle and bone disease. The CSF analysis suggested that our patient may have FTD due to NFT accumulation similar to the familial FTD patients with p.Asp395Gly VCP recently reported. Our findings suggest that a genetic search for the pathogenic variants of VCP should be considered not only for familial FTD, but also for patients with sporadic FTD, even in the absence of comorbid muscle or bone disease.

**Supplementary Information:**

The online version contains supplementary material available at 10.1186/s12883-022-02951-4.

## Background

Following Alzheimer’s disease (AD), frontotemporal dementia (FTD) is the second most common neurodegenerative disorder in patients with onset of dementia before 65 years of age [1]. FTD is a clinically, pathologically, and genetically heterogeneous syndrome that presents with personality and behavioral changes reflecting dysfunction of the frontal and temporal lobes [2]. So far, several causative genes for FTD have been identified, with hexanucleotide repeat expansion in *C9ORF72* being the most common genetic cause of both familial (~ 25%) and sporadic (~ 5%) FTD [1, 3]. Familial FTD accounts for approximately 30–50% of all FTD cases in Euro-American countries; however, the frequency of familial FTD is reportedly relatively rare in Asian regions, including India, Indonesia, Japan, Taiwan, and Philippines, where family history of FTD spectrum disorders was reported in 5.5% of FTD patients [1, 4]. This might be due to the genetic differences of the frequency of FTD patients carrying expanded repeats in *C9ORF72*, which is substantially lower in the Japanese series than in Euro-American series [3].

Variants in the *valosin-containing protein (VCP)* gene were identified as one of the causes for inclusion body myopathy (IBM) associated with Paget disease of the bone (PDB) and FTD (IBMPFD) [5]. Subsequently, pathogenic variants in *VCP* have been shown to contribute various neurodegenerative disorders, such as amyotrophic lateral sclerosis, Parkinson’s disease, and Charcot–Marie–Tooth disease [5–7]. The phenotypes of patients with *VCP* pathogenic variants are highly diverse; approximately 90% of the patients have IBM, 30–40% have PDB, and 15–30% have FTD, with overlapping symptoms. On the other hand, only 2–3% of the patients with *VCP* pathogenic variants had FTD alone phenotype [5, 8]. Thus, a small number of the previously reported patients with pathogenic variants in *VCP* had only FTD symptoms without IBM and PDB [1, 5, 8–12]. In the present study, we report a case of likely sporadic FTD in a Japanese patient carrying the pathogenic variant in *VCP* (p.Asp395Gly), without evidence of IBM or PDB, more than 15 years after the onset of FTD symptoms.

## Case presentation

**Fig. 1:**
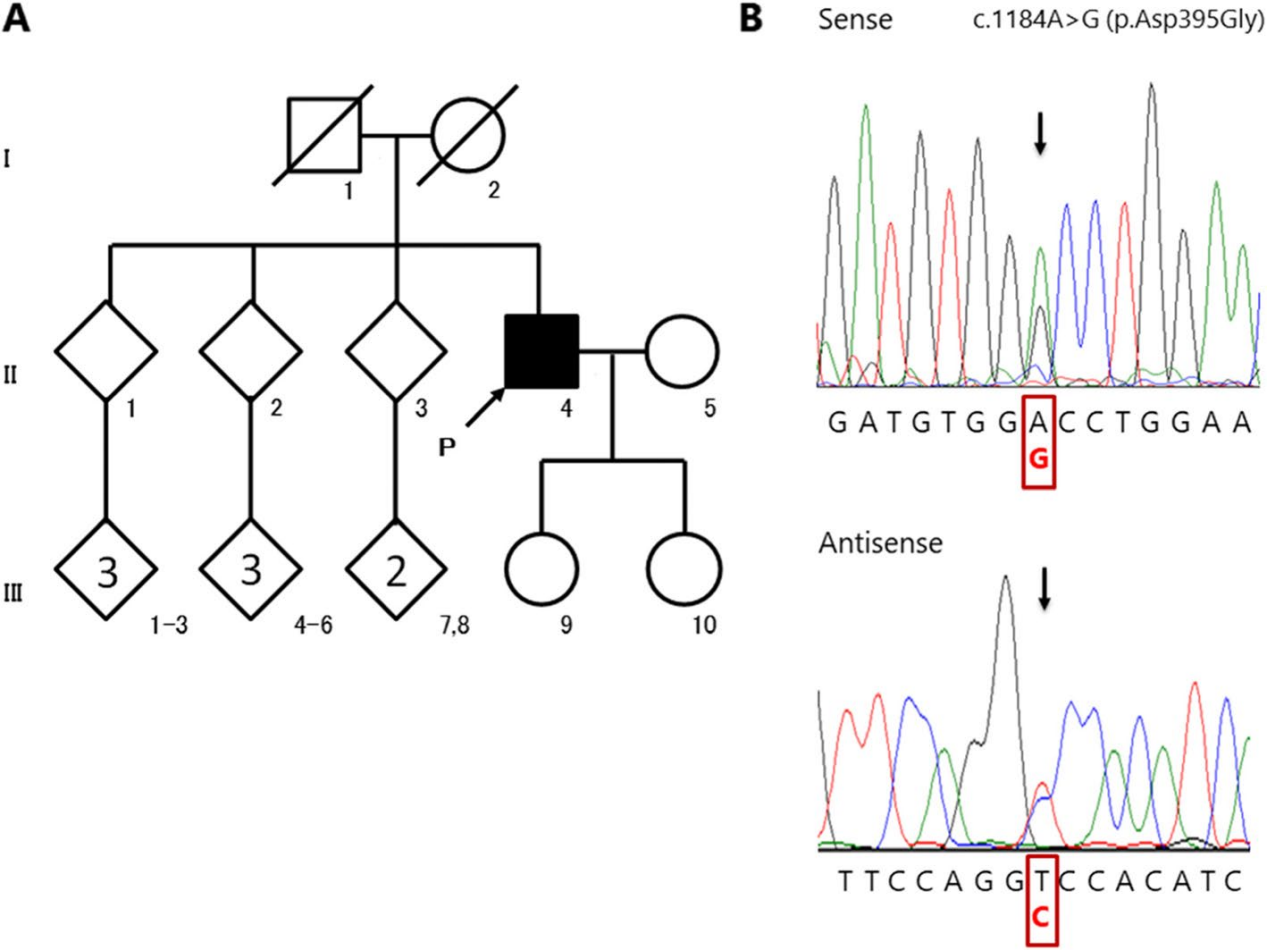
Pedigree chart and electropherograms in the patient with FTD. **a** Pedigree chart of the patient with FTD. The patient with FTD is indicated by the filled symbol. Unaffected individuals are indicated by open symbols. Slashed symbols indicate deceased individuals. Squares denote the male family members and circles denote the female family members. **b** Electropherograms of the heterozygous *VCP* c.1184A > G (p.Asp395Gly) pathogenic variant (arrow) in the patient (II-4). The use of sense (upper column) and antisense (lower column) primers revealed heterozygous *VCP* c.1184A > G variant in the patient. FTD: frontotemporal dementia

The patient was a 62-year-old, right-handed Japanese man. His pedigree chart is depicted in Fig. 1a. Written informed consent for genetic analysis and publication of this report was obtained from the patient’s wife. His past medical history was unremarkable and no similar disease was noted in his family. He graduated from university at the age of 22 years and worked as a civil servant employed by prefectural governments thereafter. He presented with depressive mood due to a heavy burden of work and visited a psychiatric clinic at 37 years of age. He was diagnosed with depression and was administered antidepressant medication. Since then, he repeatedly requested temporary leaves of absence and lacked the motivation to work and to perform household chores, however, his motivation for entertainment and hobbies did not diminish. This led to the consideration of atypical depression rather than typical depression, which is characterized by a persistent decrease in motivation. At the age of 45 years, he tended to indulge in self-centered behaviors, such as visiting a movie theater or playing a Japanese gambling machine without permission, while pretending to go to work. From around the age of 50 years, self-centered behaviors such as absenteeism and returning home from work without permission worsened, followed by executive dysfunction associated with planning and doing work. He was absent from work due to these behaviors and was referred to our hospital at the age of 52 years.

**Fig. 2:**
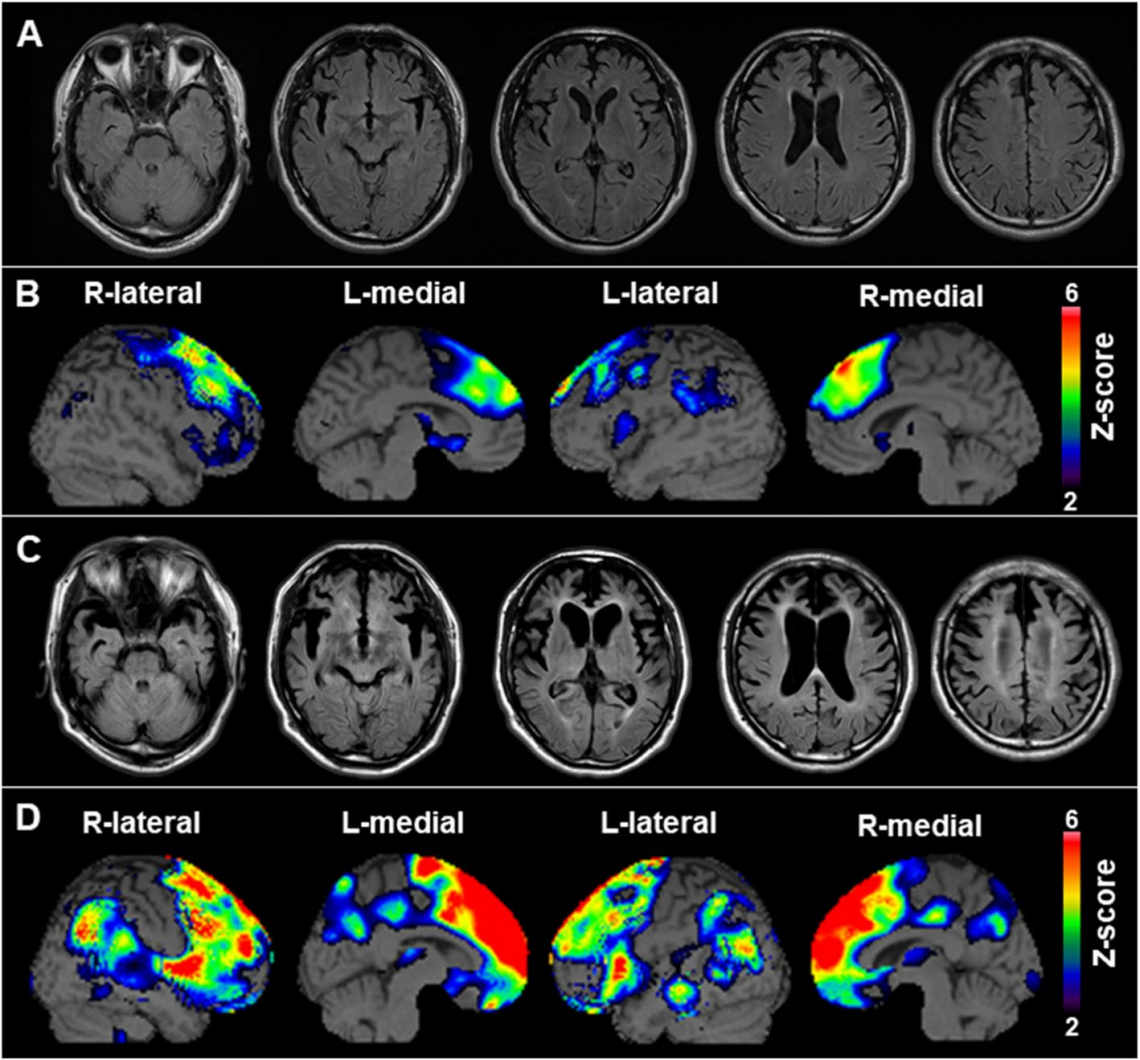
Neuroradiological findings. **a** Transverse fluid-attenuated inversion recovery (FLAIR) magnetic resonance imaging (MRI) shows bilateral frontal lobe atrophy. **b** Brain perfusion single-photon emission computed tomography (SPECT) easy Z-score Imaging System (eZIS) analysis (FUJIFILM Toyama Chemical Co., Ltd., Tokyo, Japan) shows relative hypoperfusion in the frontal lobe. The color scale for the Z score is shown in the right part of the figure. The colored areas represent Z scores > 2. **c** Transverse FLAIR MRI performed 10 years later shows remarkable bilateral frontal and temporal lobe atrophy. **d** eZIS analysis performed 10 years later reveals remarkable hypoperfusion in the frontal and parietal lobes

At the initial visit, the patient maintained good manners, although he was not lively and lacked seriousness for his situation and improvement of his condition. His Mini-Mental State Examination score was 25, failing in the domains of orientation to place, attention/ calculations, recall, and repetition. Moreover, his frontal lobe dysfunction was revealed by Frontal Assessment Battery [13, 14], a short bedside cognitive and behavioral battery to assess frontal lobe functions (score was 12/18). The results of other neurological examinations were normal. Magnetic resonance imaging (MRI) revealed bilateral frontal lobe atrophy (Fig. 2a). Technetium-99 m ethyl cysteinate dimer ([^99m^Tc]ECD) single-photon emission computed tomography (SPECT) revealed bilateral frontal lobe hypoperfusion (Fig. 2b). The patient was diagnosed with a behavioral variant of FTD based on Rascovsky et al.’s diagnostic criteria [2] and had to leave work. He was admitted to a psychiatric hospital at the age of 54 due to prominent self-centered behavior, overeating, irritability, and disinhibition.

The FTD progressed over the years, and by the age of 62 years, he had almost no speech and had a remarkable decrease in spontaneity. He needed help to perform activities of daily living but was able to eat and walk by himself. Muscle weakness and atrophy of the extremities were not observed. MRI revealed substantial atrophy in the frontal and temporal lobes and periventricular leukoaraiosis (Fig. 2c). [^99m^Tc]ECD SPECT revealed bilateral frontal and parietal lobe hypoperfusion (Fig. 2d). [^11^C]Pittsburgh compound B-positron emission tomography was negative for the presence of amyloid. His laboratory data, including the creatine kinase level, were normal. The concentrations of cerebrospinal fluid (CSF) total tau and phosphorylated tau proteins were 389 pg/mL and 53.2 pg/mL (cut-off: 50 pg/mL), respectively.

There were no signs of bone involvement (e.g., elevated serum alkaline phosphatase levels). Computed tomography of the trunk and limbs did not reveal muscle atrophy. Bone scintigraphy did not identify prominent bone lesions. There were no myopathic features or inclusion bodies on the right rectus femoris muscle biopsy (Supplementary Fig).

### Genetic analyses

Whole-exome sequencing analysis was performed for the proband (II-4 in Fig. 1a) as described in a previous study [15] and revealed a heterozygous variant (c.1184A > G), p.Asp395Gly, in *VCP* (NM_007126.5), followed by confirmation by Sanger sequencing (Fig. 1b). Expanded repeats in *C9ORF72* were not detected by repeat-primed PCR analysis [3]. Non-synonymous or splice-site variants in other genes relevant to FTD (*MAPT, GRN, TARDBP, FUS, TBK1, CHMP2B*, and *SQSTM1*) as well as those relevant to AD (*APP, PSEN1*, and *PSEN2*) [1] which were either rare (< 1% minor allele frequency) or absent in population databases were not detected on the basis of the whole-exome sequencing data [15]. The *VCP* variant c.1184A > G, p.Asp395Gly, which has been reported as pathogenic in ClinVar and Human Gene Mutation Database, was also predicted to be pathogenic according to the American College of Medical Genetics and Genomics guidelines (PS1 + PS3) [16] (described in Supplementary material).

## Discussion and conclusions

In the present study, we report a Japanese patient carrying p.Asp395Gly *VCP*, which manifested as likely sporadic FTD devoid of associated muscle or bone involvement. Relatedly, a recent study reported that four patients in two pedigrees with familial FTD carrying p.Asp395Gly *VCP* had typical FTD symptoms but did not display IBM or PDB [12]. Unfortunately, in the current study, genetic tests for the other relatives, including the patient’s parents, could not be performed. The patient’s father died due to myocardial infarction at 87 years of age and his mother of stomach cancer at age 40. Neither had a history of FTD, muscle weakness, or bone-related symptoms. Moreover, none of his three siblings (aged above 70 years), or their children, developed the disease. As such, there may be a possibility that the parents, especially his mother, carried the *VCP* pathogenic variant. Alternatively, the FTD in this case may have been caused by a de novo mutation, although we could not arrive at a definite conclusion. Despite these limitations, this study contributes to further establish the genotype-phenotype correlation of p.Asp395Gly *VCP* with pure FTD in addition to the previous report [12].

Some cases of pure FTD without IBM or PDB have been reported in FTD patients carrying pathogenic variants linked to FTLD-VCP. Of these, most are familial, whereas very few sporadic cases have been reported [1, 5, 8–12]. Our patient’s age of 62 years was above the mean age of onset for VCP-related IBM and PDB (40.4 ± 10.0 and 48.2 ± 10.9 years, respectively) [5], and the patient was therefore likely to have pure FTD. Furthermore, our patient carrying p.Asp395Gly *VCP* was afflicted by likely sporadic FTD, which may suggest the need for screening for pathogenic *VCP* variants in the Japanese FTD patients, regardless of the family history or the absence of the features of IBM or PDB.

In general, previously identified pathogenic variants in *VCP* other than p.Asp395Gly are associated with frontotemporal lobar degeneration with TDP-43 inclusions (FTLD-TDP) pathologically [8] and cause a hereditary disease called multisystem proteinopathy that affects various organs including the nervous system, skeletal muscle, and bone [5]. On the contrary, p.Asp395Gly *VCP* was recently reported to cause FTD with tauopathy characterized by the accumulation of neuronal vacuoles and neurofibrillary tau tangles (NFT) and not FTLD-TDP [12]. Notably, in this case, the CSF phosphorylated tau level was mildly increased unlike in typical FTD patients. Therefore, our patient may also have FTD due to NFT accumulation similar to the FTD patients with p.Asp395Gly *VCP*, although detailed CSF analysis including phosphorylated tau level was not described in the previous report [12].

Interestingly, this patient presented with symptoms of atypical depression before the development of behavioral abnormalities, unlike the previous studies where patients with p.Asp395Gly *VCP* had only typical FTD symptoms. The symptoms of atypical depression, in this case, may have been connected with very mild frontal lobe dysfunction, such as apathy and disinhibition. Other studies have reported that patients with progressive muscular atrophy and psychiatric symptoms, such as depression and anxiety, who subsequently developed FTD after 12 years from the onset, had the *VCP* pathogenic variant (p.Arg155His) [17]. Thus, the atypical depression preceding FTD in this case may have been associated with p.Asp395Gly *VCP*. The clinical phenotypes of cases with the *VCP* pathogenic variant (p.Asp395Gly) need to be further investigated in a wide variety of populations.

In conclusion, p.Asp395Gly *VCP* was identified in a patient with likely sporadic FTD without muscle and bone involvement. Our findings suggest that FTD with p.Asp395Gly *VCP*, akin to other hereditary FTDs, may present with psychiatric disorders preceding the development of FTD. In addition, *VCP* variants should be considered in patients diagnosed with sporadic pure FTD, even in the absence of IBM or PDB. Further studies are needed to clarify the clinical phenotype of patients with p.Asp395Gly *VCP*.

## Supplementary Information

**Additional file 1: Supplementary Figure.** Quadriceps muscle biopsy of the patient. There were no myopathic features or inclusion bodies. (a) (b) Hematoxylin and eosin staining (Scale bar: 100 μm) (c) Modified Gomori trichrome staining (Scale bar: 100 μm) (d) Nicotinamide adenine dinucleotide dehydrogenase-tetrazolium reductase staining (Scale bar: 100 μm)

**Additional file 2.**

## Data Availability

The datasets generated during the current study are available from the corresponding author on reasonable request.

## Abbreviations

[^99 m^Tc]ECD: Technetium-99-ethyl cysteinate dimer
CSF: Cerebrospinal fluid
FTD: Frontotemporal dementia
FTLD-TDP: Frontotemporal lobar degeneration with TDP-43 inclusions
IBM: Inclusion body myopathy
IBMPFD: Inclusion body myopathy associated with Paget disease of the bone and frontotemporal dementia
MRI: Magnetic resonance imaging
NFT: Neurofibrillary tau tangles
PDB: Paget disease of the bone
SPECT: Single-photon emission computed tomography
TDP-43: TAR DNA-binding protein of 43 kDa
VCP: Valosin-containing protein
