# Supplementary figures and images for: Valosin-containing protein Asp395Gly mutation in a patient with frontotemporal dementia: a case report
Source: BMC Neurol. 2022 Nov 3;22:406. doi: 10.1186/s12883-022-02951-4 (PMC9632072; doi:10.1186/s12883-022-02951-4)

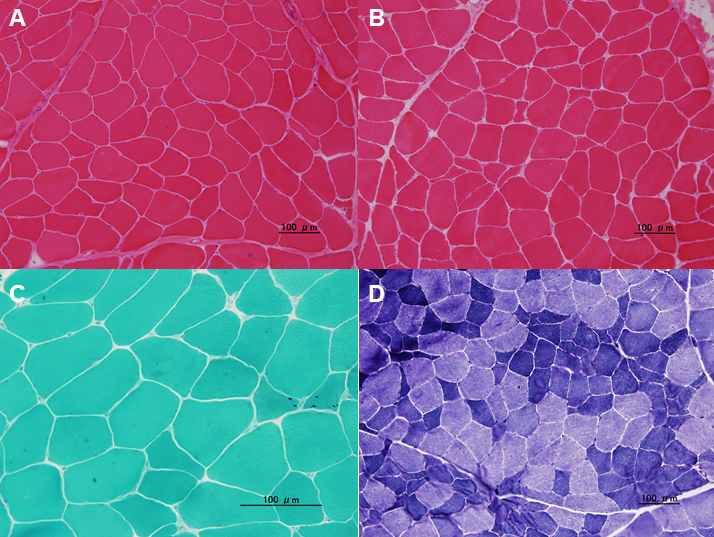

Supplement: Supplementary file 1 — Additional file 1: Supplementary Figure. Quadriceps muscle biopsy of the patient. There were no myopathic features or inclusion bodies. (a) (b) Hematoxylin and eosin staining (Scale bar: 100 μm) (c) Modified Gomori trichrome staining (Scale bar: 100 μm) (d) Nicotinamide adenine dinucleotide dehydrogenase-tetrazolium reductase staining (Scale bar: 100 μm) [file 12883_2022_2951_MOESM1_ESM.tif]
