# Supplementary material for: Valosin-containing protein Asp395Gly mutation in a patient with frontotemporal dementia: a case report
Source: BMC Neurol. 2022 Nov 3;22:406. doi: 10.1186/s12883-022-02951-4 (PMC9632072; doi:10.1186/s12883-022-02951-4)
Supplement: Supplementary file 2 — Additional file 2. [file 12883_2022_2951_MOESM2_ESM.docx]

**Supplementary material**

The *VCP* variant c.1184A>G, p.Asp395Gly, was predicted to be pathogenic according to the American College of Medical Genetics and Genomics guidelines (PS1*+PS3**).

*PS1

Same amino acid change as a previously established pathogenic variant regardless of nucleotide change [1]

**PS3

Well-established *in vitro* or *in vivo* functional studies supportive of a damaging effect on the gene or gene product [1]

[1] Darwich NF, Phan JM, Kim B, Suh E, Papatriantafyllou JD, Changolkar L, et al (2020) Autosomal dominant VCP hypomorph mutation impairs disaggregation of PHF-tau. Science 370:eaay8826. <https://doi.org/10.1126/science.aay8826>
